# Complete Genome Sequence of vB_EcoP_PR_Kaz2018, a T7-Like Bacteriophage

**DOI:** 10.1128/MRA.01323-19

**Published:** 2019-12-05

**Authors:** Madina S. Alexyuk, Andrey P. Bogoyavlenskiy, Pavel G. Alexyuk, Yergali S. Moldakhanov, Aizhan S. Turmagambetova, Elmira I. Anarkulova, Vladimir E. Berezin

**Affiliations:** a Research and Production Center for Microbiology and Virology, Laboratory of Antiviral Protection, Almaty, Kazakhstan; Queens College

## Abstract

Here, we describe the complete genome sequence of the Escherichia coli bacteriophage vB_EcoP_PR_Kaz2018, isolated from a water sample. vB_EcoP_PR_Kaz2018 is a linear double-stranded DNA T7-like podophage with a genome of 39,704 bp containing 45 predicted open reading frames (ORFs).

## ANNOUNCEMENT

The Escherichia coli bacteriophage vB_EcoP_PR_Kaz2018 was isolated from a water sample and is capable of infecting encapsulated Escherichia coli expressing the K1 capsular antigen, a major causative agent of neonatal septicemia, sepsis, and meningitis (1, 2).

Here, we present the complete genome sequence of this bacteriophage, isolated from a freshwater sample collected from the Pravyi Esentai River near the city of Almaty, Kazakhstan. The phage was found to be using as its host a clinical isolate of Escherichia coli. The bacterium was cultivated on MacConkey broth at 37°C. The virulence factors associated with the E. coli isolate were not investigated in this study. A phage enrichment method was used to isolate the bacteriophage against the host bacterial strain. After incubation, the cocultivation solution was centrifuged at 6,000 rpm, followed by filtration of the supernatant with a 0.45-μm membrane. The filtrate was tested for phage activity using a spot test and the double-agar overlay method. A single phage plaque was harvested from the agar overlay plate. All of the plaque isolation steps were repeated three times for phage purification.

The genomic DNA was extracted from the phage lysate of the E. coli isolate according to the guide for the PureLink viral DNA/RNA minikit (Thermo Fisher Scientific, USA). The DNA library was prepared using the Nextera XT DNA sample preparation kit (Illumina). Whole-genome sequencing was performed with an Illumina MiSeq sequencing platform. Low-quality reads were filtered and adapters trimmed with Trimmomatic (3) from the Genome Detective tool (4). As a result, a total of 3,133,772 paired-end reads were assembled using SPAdes 3.12.0 (5). The average read length after trimming was 286 bp. The genome was sequenced to an average depth of more than 1,000× (4, 6). The physical ends of the viral genome were identified by comparing the coverage values of the length of the complete genome of the virus vB_EcoP_PR_Kaz2018 to those of closely related viruses in the NCBI database with BLAST and ViroBlast (7, 8), namely, Enterobacter phage K1F (GenBank accession number AM084414), with 100% query coverage and 95.28% identity; Escherichia phage LM33_P1 (LT594300), with 86% query coverage and 95.22% identity; *Escherichia* phage PE3-1 (KJ748011), with 85% query coverage and 95.13% identity; and *Escherichia* phage YZ1 (MG845865), with 84% query coverage and 94.73% identity.

The complete genome of phage vB_EcoP_PR_Kaz2018 is a linear double-stranded DNA (dsDNA) of 39,704 bp, with 178-bp-long terminal repeats. The GC content of the genome was 49.8%. Gene prediction was done using GeneMark and PHAST (9, 10). A total of 45 putative genes were predicted in the complete genome of the vB_EcoP_PR_Kaz2018 phage. An analysis of the phage open reading frames (ORFs) using a BLAST search showed that 24 ORFs correspond to proteins with a specific function. The remaining ORFs correspond to hypothetical viral proteins. This genome further increases the known diversity of bacteriophages capable of infecting E. coli.

### Data availability.

The complete genome sequence of the *Escherichia* phage vB_EcoP_PR_Kaz2018 was deposited in GenBank under the accession number MN510331. Raw sequence reads are available under BioProject accession number PRJNA574554.
